# Cuprotosis-Related Genes: Predicting Prognosis and Immunotherapy Sensitivity in Pancreatic Cancer Patients

**DOI:** 10.1155/2022/2363043

**Published:** 2022-09-09

**Authors:** Yingkun Xu, Han Li, Ailin Lan, Qiulin Wu, Zhenrong Tang, Dan Shu, Zhaofu Tan, Xin Liu, Yang Liu, Shengchun Liu

**Affiliations:** Department of Breast and Thyroid Surgery, The First Affiliated Hospital of Chongqing Medical University, Chongqing 400042, China

## Abstract

Based on TCGA, GTEx, and TIMER databases and various bioinformatics analysis methods, the potential biological roles of cuprotosis-related genes in pancreatic cancer were deeply explored, and a predictive model for pancreatic cancer patients was constructed. We downloaded the RNA-Seq data and clinicopathological and predictive data of 179 pancreatic cancer tissues and 332 adjacent normal tissues from TCGA and GTEx databases. The differential expression of cuprotosis-related genes in pancreatic cancer tissue and adjacent normal tissue was analyzed, and the LASSO regression algorithm was used to construct a prediction model and verify the validity of the model prediction. Based on the LASSO regression algorithm, a predictive model composed of three genes LIPT1, LIAS, and DLAT was screened. The corresponding survival curves showed that the constructed prediction model could significantly distinguish the prognosis of pancreatic cancer patients, and the prognosis of patients in the high-risk group was worse (*P* = 0.00557). The ROC curve showed that the area under the curve of the predictive model for predicting the 4-, 5-, and 6-year survival rates in pancreatic cancer was 0.816, 0.836, and 0.956, respectively. The AUC value of this risk model was significantly higher than 0.7, which could more accurately predict the prognosis of pancreatic cancer patients. This study determined a risk-scoring model of cuprotosis-related genes, which can provide an essential basis for judging the prognosis of pancreatic cancer patients.

## 1. Introduction

Pancreatic cancer is one of the most malignant cancers among digestive tract cancers and has the characteristics of rapid progression and high mortality [1, 2]. It is predicted that by 2030, pancreatic cancer will become the second most fatal cancer in the world [3]. It is related to alcohol consumption, smoking, a high-fat and high-protein diet, excessive coffee consumption, environmental pollution, and genetic factors. Pancreatic cancer is common in patients with diabetes or chronic pancreatitis, and approximately 90% are pancreatic ductal adenocarcinomas [4, 5]. Due to its insidious onset, no typical symptoms in the early stage, and intense infiltration and metastasis characteristics, most patients have progressed to the middle and late stages of the disease when they visit a doctor, missing the best period for radical surgical treatment, and more than 80% of patients are unresectable [6]. At the same time, it shows extensive tolerance to radiotherapy and chemotherapy. It is not sensitive to immunotherapy, resulting in poor treatment effects, so the prognosis is poor, and the 5-year survival rate is only 10% [7–10]. In addition to imaging methods such as CT and MRI, tumor markers such as CEA and CA199 are also commonly used in diagnosing pancreatic cancer [11, 12]. However, these markers have poor sensitivity and specificity in diagnosing pancreatic cancer and usually do not increase significantly in the early stage. By the time it is abnormally elevated, the disease is generally in the middle and late stages [13, 14]. The etiology and pathogenesis of pancreatic cancer are not yet fully understood. Therefore, there is an urgent need to find biomarkers and therapeutic targets for pancreatic cancer to improve the quality of life and overall survival of pancreatic cancer patients.

Copper is a common metal element and a transition element with redox activity. Under conventional chemical reactions and physiological conditions, reduced Cu ^+^ can be transformed into oxidized Cu^2+^ [15]. Copper ions participate in various biochemical reactions by donating or accepting electrons [16]. Copper ions can be combined with multiple proteins or enzymes as cofactors or structural components and participate in the regulation of numerous physiological processes such as energy metabolism, mitochondrial respiration, and antioxidation. The content of copper ions maintains a dynamic balance, and the imbalance can lead to oxidative stress and abnormal autophagy [17, 18], thereby inducing various copper or copper ion-related diseases. On March 17, 2022, Tsvetkov et al. first proposed a copper-dependent, new cell death mode named “cuprotosis” [19, 20], which is different from other known cell death modes such as apoptosis, pyroptosis, and necroptosis, while analogous to zinc death and ferroptosis, is a type of metal ion-induced regulated cell death [21]. Cu^2+^ directly binds to fatty acylated components of the tricarboxylic acid cycle in mitochondrial respiration, leading to fatty acylated protein aggregation and subsequent downregulation of iron-sulfurclusterin-inducing cells independent of the apoptotic pathway to dying. Several studies have suggested that targeting copper metabolism can improve the sensitivity of tumor chemoprevention and anticancer drugs [22–24]. Furthermore, excess copper leads to the loss of FDX1-dependent Fe-S cluster proteins, whereas inhibiting Fe-S cluster formation significantly reduces mitochondrial fatty acylation. It is suggested to utilize the unique role of fatty acylated protein modification in copper toxicity and explore the potential application of copper toxicity-based targeted intervention [19, 25–29]. Therefore, cuprotosis can be a potential intervention target for harm or diseases related to copper metabolism dysfunction, and at the same time, it may provide a new antitumor method.

Due to the lack of comprehensive exploration of cuprotosis-related genes in pancreatic cancer, we used various bioinformatics tools to explore the potential biological roles and immune correlations of cuprotosis-related genes in pancreatic cancer.

## 2. Materials and Methods

### 2.1. Data Collection

In this study, we obtained RNA-Seq data, clinicopathological, and prognostic information of pancreatic cancer samples and normal pancreatic samples through The Cancer Genome Atlas (TCGA) database (https://portal.gdc.com). The GTEx database can help researchers understand genetic susceptibility to disease and will serve as a resource database and tissue repository for many future studies. In addition, to increase the RNA-Seq data of adjacent normal tissue samples, we also obtained more RNA-Seq data of adjacent normal tissue samples through the Genotype-Tissue Expression (GTEx) database (https://gtexportal.org/home/) [30, 31].

### 2.2. Selection of Cuprotosis-Related Genes

In a research paper by Tsvetkov et al. titled “Copper induces cell death by targeting lipoylated TCA cycle proteins”, the main findings show that copper toxicity occurs by a mechanism distinct from all other known mechanisms of cell death, including apoptosis, ferroptosis, pyroptosis, and necroptosis, and termed this previously uncharacterized cell death mechanism cuprotosis [19]. The researchers have mainly discovered ten regulatory genes related to the cuprotosis metabolic pathway, including seven positive regulatory genes FDX1, LIPT1, LIAS, DLD, DLAT, PDHA1, and PDHB, and three negative regulatory genes MTF1, GLS, and CDKN2A [19]. It will be a future research focus for researchers to explore the specific roles of these genes in the process of cuprotosis and the effectiveness of copper toxicity in treating cancer.

### 2.3. Data Processing

In this study, we downloaded the STAR-counts data and clinical information of the pancreatic cancer dataset from the TCGA database (https://portal.gdc.com), from which we extracted the data in TPM format. We then normalized it to log2 (TPM+1), ultimately retaining samples with RNA-Seq data and clinical information. LASSO regression algorithm was used to construct the risk model, and a 10-foldcross-validation was used. The above analysis was performed using the *R* software “glmnet” package. The log rank was used to test the KM survival analysis to compare the survival difference between the above two or more groups, and the timeROC analysis was performed to determine the accuracy of the risk model. The correlation analysis between the risk model and immune cell infiltration was performed by the *R* software package “ggstatsplot.” In addition, we used the *R* software package “ConsensusClusterPlus” for consistency analysis, the maximum number of clusters was 6, and 80% of the total samples were drawn with 100 repetitions. The *R* package “pheatmap” analyzed heat maps for cluster analysis.” All the above analytical methods and *R* packages were performed using *R* software v4.0.3. *P* < 0.05 was considered statistically significant.

### 2.4. GSCA Website

Developed by Prof. Guo Anyuan's research group at Huazhong University of Science and Technology, GSCA provides various services to perform genomics and immunogenomics analysis (http://bioinfo.life.hust.edu.cn/GSCA/#/). GSCA integrates 10,000 genomic data from 33 cancer types from TCGA and more than 750 small molecule drugs from GDSC and CTRP. Combining clinical information and small molecule drugs, users can mine candidate biomarkers and valuable drugs for better experimental design and further clinical trials [32]. In this study, we used the GSCA to explore the genetic variation and drug sensitivity of cuprotosis-related genes in pancreatic cancer.

### 2.5. TIMER Database

TIMER is a website tool established by Professor *Xiaole* Shirley Liu of Harvard University (http://timer.cistrome.org/). TIMER uses deconvolution to infer the abundance of tumor-infiltrating immune cells (B cells, CD4+ T cells, CD8+ T cells, neutrophils, macrophages, and dendritic cells) from gene expression profiles of samples from different cancer types in TCGA, enabling comprehensive and flexible tumor-infiltrating immune cells were analyzed and visualized [33–35]. This study used TIMER to explore the association between cuprotosis-related genes and immune cell infiltration in pancreatic cancer.

### 2.6. Statistics

In this study, we used *R* software v4.0.3 for statistical analysis. The Wilcoxon test was used to compare the mRNA expression differences of cuprotosis-related genes in the two groups of samples. In addition, the corresponding data were analyzed using statistical analysis tools from multiple online databases. *P* < 0.05 was considered statistically significant.

## 3. Results

### 3.1. The Expression and Prognosis of Cuprotosis-Related Genes in Pancreatic Cancer

**Figure 1:**
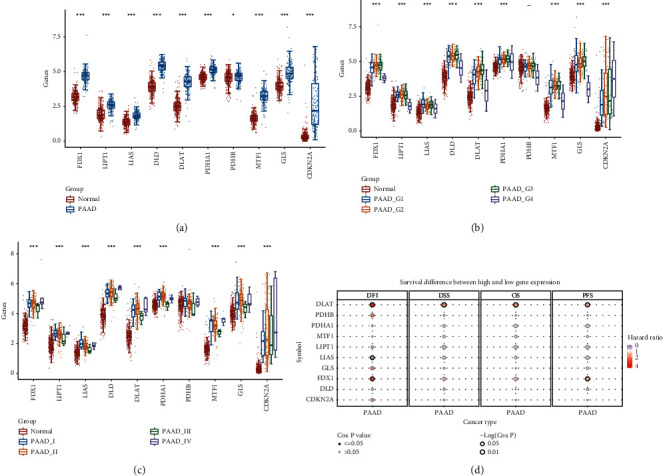
mRNA expression and clinical prognosis of cuprotosis-related genes in pancreatic cancer. (a) Combined with TCGA and GTEx databases, the boxplot shows the mRNA expression of cuprotosis-related genes in pancreatic cancer and normal pancreatic tissue. (b), (c) Boxplots showing the mRNA expression of cuprotosis-related genes in different grades and TNM stages of pancreatic cancer. (d) Bubble plot showing DFI, DSS, OS, and PFS of cuprotosis-related genes in pancreatic cancer. ^*∗*^*P* < 0.05, ^*∗∗*^*P* < 0.01, and ^*∗∗∗*^*P* < 0.001.

To explore whether there are differences in the mRNA expression of these ten cuprotosis-related genes in pancreatic cancer, we extracted the mRNA expression data of these ten cuprotosis-related genes from TCGA database and the GTEx database. We drew a boxplot to visualize the differences in the mRNA expression of these ten cuprotosis-related genes (Figure 1(a)). The results showed that the mRNA expression of all cuprotosis-related genes was significantly different between pancreatic cancer samples and normal pancreatic samples and showed abnormally high expression in pancreatic cancer samples. Then, we drew corresponding boxplots to display them separately to explore whether these ten cuprotosis-related genes differ in different grades and TNM stages of pancreatic cancer (Figures 1(b)-1(c)). The results showed that the mRNA expressions of 10 cuprotosis-related genes were upregulated to varying degrees with the progression of pancreatic cancer. In addition, to explore the prognostic value of these ten cuprotosis-related genes in pancreatic cancer, we drew corresponding bubble charts to show the DFI, DSS, OS, and PFS of these ten cuprotosis-related genes in pancreatic cancer (Figure 1(d)). The results showed that DLAT, FDX1, and LIAS were significantly correlated with DFI in pancreatic cancer patients, and DLAT was significantly correlated with DFI, DSS, OS, and PFS of pancreatic cancer.

### 3.2. Gene Variation and Drug Sensitivity of Cuprotosis-Related Genes in Pancreatic Cancer

**Figure 2:**
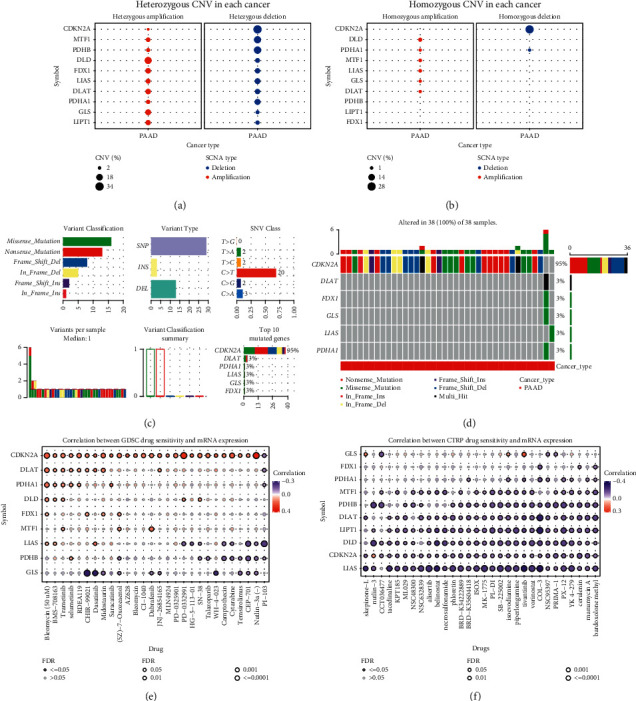
Gene variation and drug sensitivity of cuprotosis-related genes in pancreatic cancer. (a), (b) Bubble plots showing CNVs of cuprotosis-related genes in pancreatic cancer. (c), (d) The histograms and heat maps show the SNVs of cuprotosis-related genes in pancreatic cancer. (e), (f) Bubble plots showing the correlation between cuprotosis-related gene mRNA expression and sensitivity to multiple anticancer drugs based on GDSC and CTRP databases. Blue bubbles represent negative correlations, red bubbles represent positive correlations, and the darker the color, the higher the correlation. Bubble size was positively correlated with FDR significance. Black outline boxes indicate FDR ≤0.05.

To explore the genetic variation of these ten cuprotosis-related genes in pancreatic cancer, we drew the corresponding bubble chart, histogram, and heat map. After CNV analysis in pancreatic cancer for these ten cuprotosis-related genes, we found that CDKN2A, MTF1, PDHB, DLD, and FDX1 genes had higher CNVs in pancreatic cancer (Figures 2(a)–2(b)). Subsequently, we explored the SNV situation of these ten cuprotosis-related genes in pancreatic cancer, and the results showed that CDKN2A had different types of SNVs in 95% of pancreatic cancer samples (Figures 2(c)–2(d)). Furthermore, based on the GDSC and CTRP databases, we explored the sensitivity of these ten cuprotosis-related genes to various anticancer drugs. The results showed that CDKN2A significantly correlated with the sensitivity of various anticancer drugs. Meanwhile, there was a significant negative correlation between the sensitivities of LIAS to multiple anticancer drugs (Figures 2(e)–2(f)).

### 3.3. GSEA and Co-Expression Analysis of Cuprotosis-Related Genes in Pancreatic Cancer

**Figure 3:**
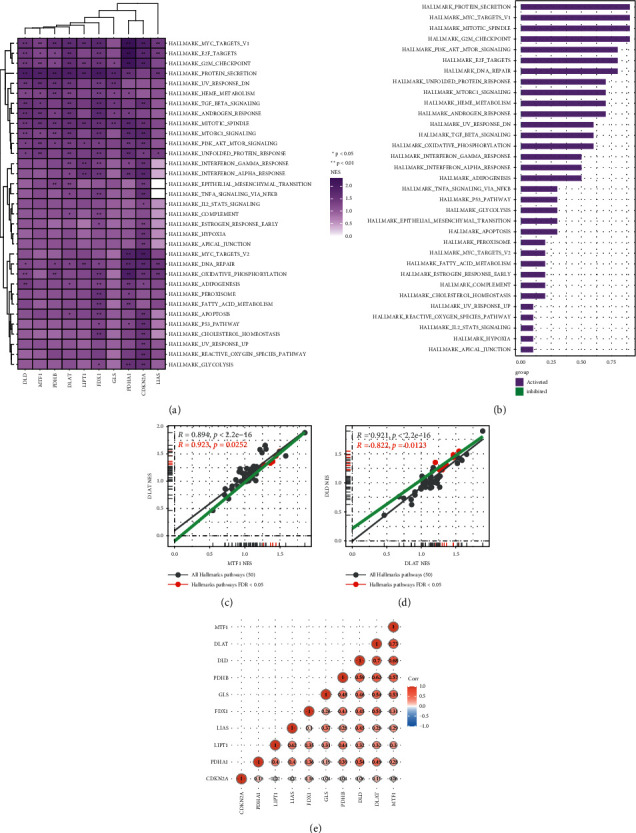
GSEA and co-expression analysis of cuprotosis-related genes in pancreatic cancer. (a), (b) The heat map and histogram show the results of the GSEA analysis of cuprotosis-related genes in pancreatic cancer. The darker the color block, the stronger the correlation between the two, ^*∗*^*P* < 0.05, ^*∗∗*^*P* < 0.01. (c), (d) Scatter plots showing similarities between DLAT and MTF1 and DLD. (e) This heat map shows the co-expression of cuprotosis-related genes in pancreatic cancer. Red represents positive correlation, and blue represents negative correlation. The shade of color represents the strength of the correlation.

To understand the biological pathways these ten cuprotosis-related genes are associated with in pancreatic cancer, we performed the GSEA analysis on these ten cuprotosis-related genes. The results of the GSEA analysis showed that these ten cuprotosis-related genes were significantly related to the abnormal activation of various biological pathways and had high similarity. The top five abnormally activated biological pathways were HALLMARK PROTEIN SECRETION, HALLMARK MYC TARGETS V1, HALLMARK MITOTIC SPINDLE, HALLMARK G2M CHECKPOINT, and HALLMARK PI3K AKT MTOR SIGNALING (Figures 3(a)–3(d)). In addition, co-expression analysis results showed a strong positive correlation between DLAT and MTF1 (Figure 3(e)). These results provide ideas for exploring these ten cuprotosis-related genes in pancreatic cancer.

### 3.4. Construction of a Risk Signature in Pancreatic Cancer Based on Cuprotosis-Related Genes

**Figure 4:**
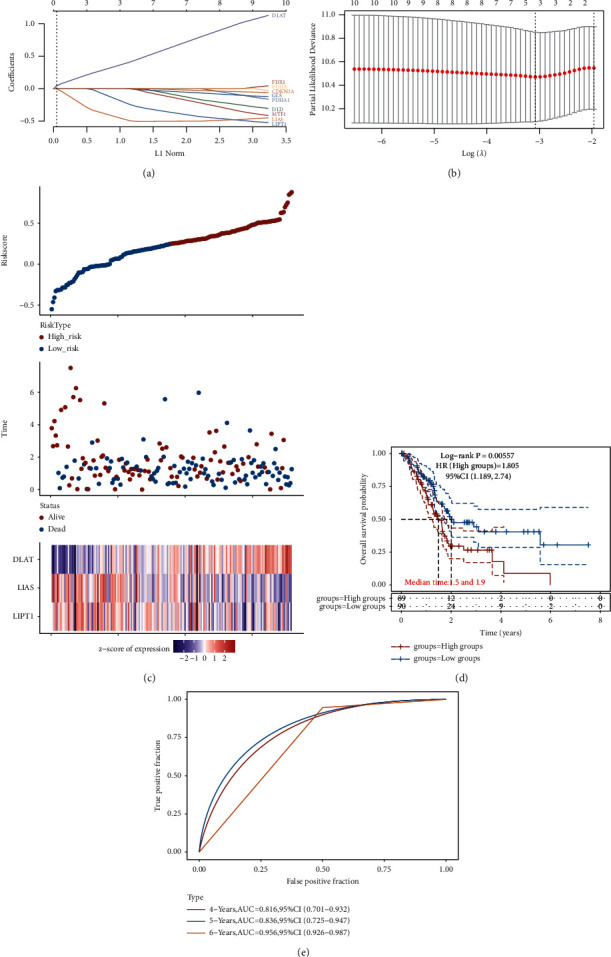
Construction of a novel risk signature in pancreatic cancer based on cuprotosis-related genes. (a), (b) Lasso coefficient values and vertical dashed lines were calculated at the best log (lambda) value, and coefficients of prognostic-related genes were displayed. (c) Risk score and survival time, and survival status of this risk signature in pancreatic cancer patients happening. (d) In the KM survival curve of the risk signature in pancreatic cancer patients, in which different groups are tested by log-rank, HR represents the risk coefficient of the high-risk group relative to the low-risk group samples. If HR > 1, the model is a dangerous model. If HR < 1, it means the model is a protection model. Median time represents the time corresponding to the survival rate of 50% in the high-risk and low-risk groups. (e) The ROC curve and AUC of this risk feature in pancreatic cancer patients at 4, 5, and 6 years. The higher the AUC value, the stronger the model's predictive ability.

To fully explore the prognostic value of these ten cuprotosis-related genes in pancreatic cancer, we used the LASSO regression algorithm to establish a new risk model composed of LIPT1, LIAS, and DLAT for pancreatic cancer patients (Figures 4(a)–4(c)). Based on the algorithm of this risk model, we can classify pancreatic cancer patients into high-risk and low-risk groups. KM survival analysis showed that the overall survival rate of the high-risk group of pancreatic cancer patients was significantly lower than that of the low-risk group (*P* = 0.00557) (Figure 4(d)). We performed ROC curve analysis to test the predictive accuracy of this cuprotosis-associated risk model in pancreatic cancer. The results showed that the four-year AUC was equal to 0.816, the five-year AUC was equal to 0.836, and the six-year AUC was equal to 0.956 (Figure 4(e)). This indicates that the cuprotosis-associated risk model has good predictive accuracy in pancreatic cancer.

The formula for calculating the risk model is as follows: Risk score = (−0.2178) *∗* LIPT1 + (−0.4758) *∗* LIAS + (0.3899) *∗*DLAT.

### 3.5. Correlation between Cuprotosis-Related Risk Signature and Immune Cell Infiltration

**Figure 5:**
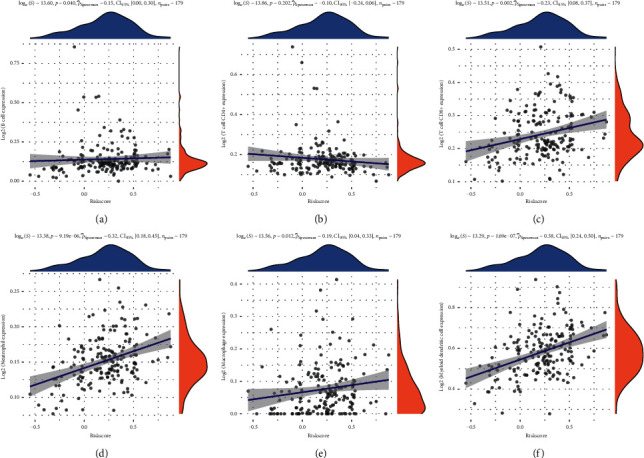
Based on the TIMER database, the Spearman correlation analysis between the cuprotosis-associated risk model and six types of immune cell infiltration in pancreatic cancer. (a) B cells, (b) CD4+ T cells, (c) CD8+ T cells, (d) neutrophil, (e) macrophage, and (f) dendritic cells. The abscissa represents the cuprotosis-related risk model score, and the ordinate represents the immune score.

To explore whether this cuprotosis-associated risk model correlates with immune cell infiltration in pancreatic cancer, we used the TIMER database to deeply explore the correlation between this cuprotosis-associated risk model and six immune cells in PAAD (B cells, CD4+ T cells, CD8+ T cells, neutrophils, macrophages, and dendritic cells). The results showed a significant positive correlation between CD8+ T cells, neutrophils, dendritic cells, and this cuprotosis-related risk model (Figures 5(a)–5(f)). These results can help us comprehensively understand the correlation between this cuprotosis-associated risk model and the six types of immune cell infiltration, providing new evidence for immunotherapy of pancreatic cancer.

### 3.6. Clustering Analysis of Pancreatic Cancer Patients Based on Cuprotosis-Related Genes

**Figure 6:**
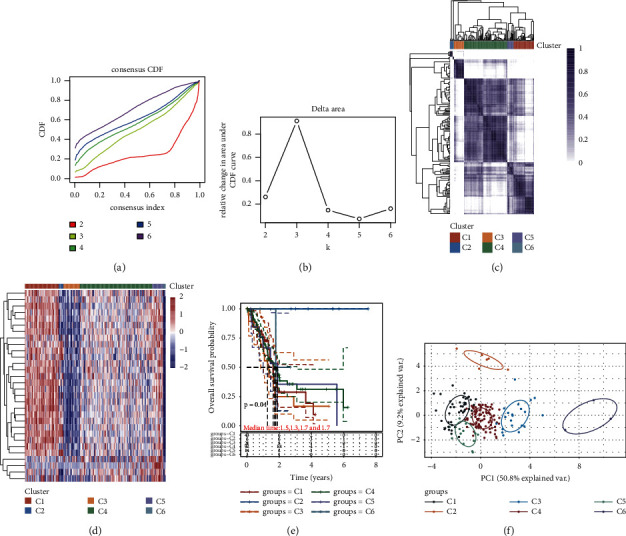
Based on TCGA database, cluster analysis was performed using cuprotosis-related genes in pancreatic cancer patients. (a), (b) CDF curve and CDF delta area curve of cluster analysis. (c) Consensus clustering matrix fork = 6. (d) Heat map showing the results of this cluster analysis. (e) KM survival curves of different subgroup samples in pancreatic cancer patients, where different groups were tested by log-rank. Median time represents the time corresponding to the survival rate of different groups at 50%. (f) Scatter plot showing the PCA results corresponding to this cluster analysis. Different colors represent different groups.

Consistent clustering, an unsupervised clustering method, is a common cancer subtype classification method that can differentiate samples into several subtypes based on different omics datasets, thereby discovering new disease subtypes. Similar to multiple previous studies [36–39], we used consensus clustering to identify subtypes in PAAD based on cuprotosis-related genes in this study (Figures 6(a)–6(c)). In this consistent clustering, we used cuprotosis-related genes to identify pancreatic cancer patients into six subtypes, and there were differences in overall survival between the different subtypes (*P* = 0.04) (Figures 6(d)–6(e)). Subsequently, the results of the PCA analysis visually showed us the outcomes of the six subtype classifications (Figure 6(f)).

### 3.7. Correlation between Cuprotosis-Related Genes and Immune Cell Infiltration in Pancreatic Cancer

**Figure 7:**
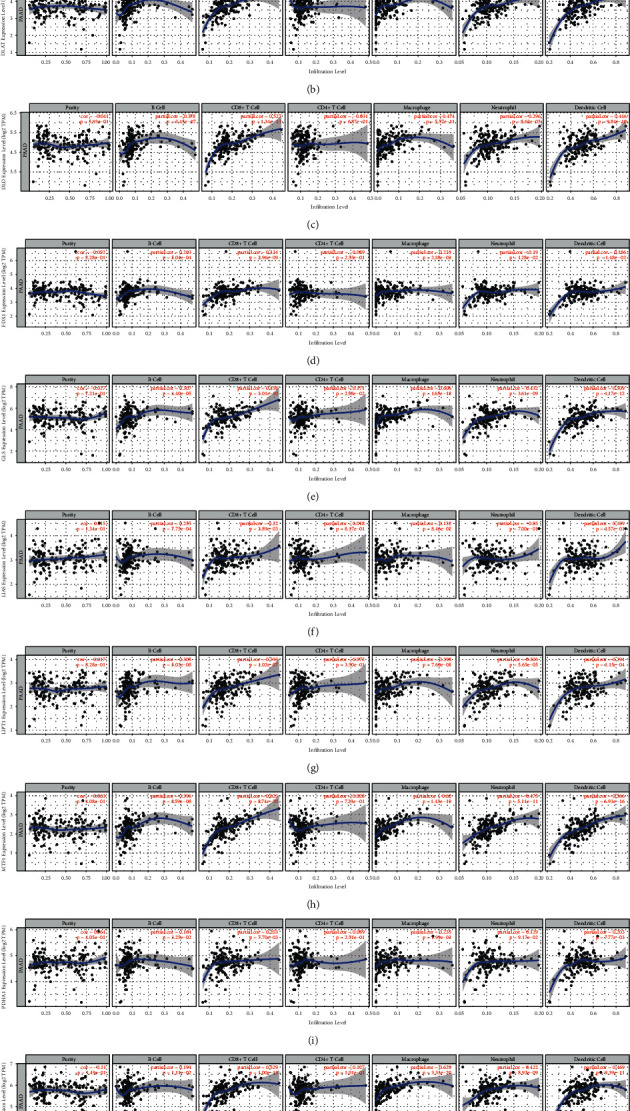
Correlation analysis between cuprotosis-related gene mRNA expression and immune cell infiltration in pancreatic cancer. (a) CDKN2A, (b) DLAT, (c) DLD, (d) FDX1, (e) GLS, (f) LIAS, (g) LIPT1, (h) MTF1, (i) PDHA1, and (j) PDHB.

To explore whether these ten cuprotosis-related genes are correlated with immune cell infiltration in pancreatic cancer, we used the TIMER database to deeply explore the correlations between these ten cuprotosis-related genes and six types of immune cells in PAAD (B cells, CD4+ T cells, CD8+ T cells, neutrophils, macrophages, and dendritic cells). The results showed a significant positive correlation between B cells, CD8+ T cells, neutrophils, macrophages, dendritic cells, and the mRNA expression of multiple cuprotosis-related genes, including DLAT, DLD, GLS, LIPT1, MTF1, and PDHB (Figures 7(a)–7(j)). Immune cell infiltration has been shown to play a critical role in tumor development and influence clinical outcomes in cancer patients. In this study, a comprehensive analysis of tumor-infiltrating immune cells will elucidate the mechanisms of immune escape in pancreatic cancer, thereby providing opportunities for developing new therapeutic strategies.

## 4. Discussion

Cuprotosis is a recently discovered form of cell death characterized by the accumulation of intracellular free copper and protein lipidation leading to cytotoxic stress, which induces cell death [19]. Multiple studies have confirmed that copper metabolism is associated with tumorigenesis, and cancer cells have higher copper requirements than normal cells [40–43]. Some cancer types express large amounts of fatty acylated mitochondrial proteins and exhibit high-intensity respiration [41, 43]. The discovery of cuprotosis is helpful for a deeper understanding of copper metabolism diseases and their underlying molecular mechanisms. It is of great value for screening related drugs for treating copper metabolism diseases. However, there is no comprehensive analysis of cuprotosis-related genes in pancreatic cancer, so we used various biological information analysis methods to deeply explore the mechanism of cuprotosis-related genes in pancreatic cancer.

At present, researchers have mainly discovered ten regulatory genes related explicitly to the cuprotosis metabolic pathway, including seven positive regulated genes (FDX1, LIPT1, LIAS, DLD, DLAT, PDHA1, and PDHB) and three negative regulatory genes (MTF1, GLS, and CDKN2A) [19]. In this study, we successfully used cuprotosis-related genes to establish a prognosis-related risk model for pancreatic cancer patients, which included three genes LIPT1, LIAS, and DLAT. Based on the cuprotosis-related risk model, clinicians can accurately identify pancreatic cancer patients with poor prognoses to achieve the purpose of precision medicine. In a previous study, Zhang et al. explored the effects of modulating FDX1 expression on LUAD cells. Their results showed that the regulation of FDX1 expression had no significant impact on LUAD cell growth, apoptosis, or cell cycle, but the knockdown of FDX1 expression significantly affected LUAD cell metabolism. Further findings showed that the knockdown of FDX1 expression mainly promoted glycolysis and fatty acid oxidation and altered amino acid metabolism [44]. Dihydrolipoamide dehydrogenase (DLD) is a homodimeric flavin-dependent enzyme that catalyzes the NAD + -dependent oxidation of dihydrolipoamide [45]. The enzyme can be modified with an Arg-Gly-Asp group, enabling it to interact with integrin-rich cancer cells, followed by “integrin-assisted endocytosis.” Previous studies have demonstrated that the expression level of DLD in various human cancer cells is significantly lower than in normal cells. This low expression is associated with poor survival outcomes across multiple cancer types, including kidney, colon, and cervical cancers [46, 47]. In addition, one study showed that DLD is closely related to ferroptosis induced by cystine deprivation or input inhibition [48].

DLAT belongs to the E2 subunit of the pyruvate dehydrogenase complex. The complex consists of three enzymes: the pyruvate dehydrogenase component, dihydrolipoamide transacetylase, and dihydrolipoamide dehydrogenase. It is the only one that converts pyruvate into acetyl-CoA after entering the mitochondria [49]. Acetyl-CoA is the primary substance entering the citric acid cycle and the starting substance for lipid synthesis [50]. Therefore, the enzyme encoded by DLAT determines to a certain extent, whether the energy-supplying substances derived from glucose can smoothly enter the citric acid cycle-oxidative phosphorylation pathway for complete hydrolysis to generate energy or supply more synthetic lipids for tumor cells [49]. It is known that pyruvate dehydrogenase kinases (PDK)-mediated phosphorylation of pyruvate dehydrogenase complex (PDC) can inhibit PDC activity, which is associated with metabolic disorders such as cancer. A new generation of PDK small molecule inhibitors can interfere with the PDK/PDC axis to enable target cells to enter mitochondrial oxidative phosphorylation instead of relying on glycolytic energy production, thereby allowing cells to return to a “quiescent” state and stop proliferation [51]. There are few studies on the role of DLAT in PDC in tumors. The available research results include Wen et al. finding that the expression of DLAT in gastric cancer cells was significantly upregulated [52], and Shan et al. found that DLAT promotes tumor cell growth by activating the pentose phosphate pathway in various tumor cells [53]. There is no research report on the relationship between DLAT expression and the prognosis of tumor patients.

As for the PDHA1 gene, there have been many reports that it has a significant prognostic value and clinical significance in various cancers. Liu et al. reported that in esophageal squamous cell carcinoma, the inhibition of PDHA1 gene expression enhanced the Warburg effect and tumor cell proliferation, migration, and drug resistance [54]. Zhong et al. found that PDHA1 is related to the degree of differentiation of esophageal squamous cell carcinoma. Its expression is higher in well-differentiated tumors, and low expression of PDHA1 is associated with poor prognosis in patients with esophageal squamous cell carcinoma [55]. Sun et al. reported that PDHA1 expression was downregulated in hepatocellular carcinoma tissue compared to adjacent liver tissue, and PDHA1 expression was correlated with tumor size, tumor differentiation, and TNM stage, but not with patient age, gender, and liver cirrhosis [56]. Overexpression of PDHA1 in liver cancer cells can reduce the activity of PDH and affect the metabolic state of liver cancer cells, thereby inhibiting the proliferation of liver cancer cells and promoting the apoptosis of liver cancer cells. In gastric cancer, the expression level of PDHA1 was significantly decreased in intestinal-type, diffuse-type, and mixed-type gastric cancer. Immunohistochemical results showed that the expression of PDHA1 protein in gastric cancer tissue was significantly lower than that in adjacent gastric mucosal tissue, and the expression of PDHA1 in poorly differentiated gastric cancer was lower than that in well-differentiated gastric cancer [57]. In ovarian cancer, low PDHA1 expression is associated with higher pathological stage and lower overall survival [58]. In addition, it has been reported that the knockdown of PDHA1 expression can inhibit the formation of prostate cancer by inhibiting adipogenesis [59].

MTF1 is a zinc finger transcription factor that promotes cell survival by activating downstream target genes, including MT1, MMP, zinc efflux protein ZnT-1, and zinc influx regulator ZIP-1 [60–63]. MTF1 is upregulated in breast, lung, and cervical cancers [64]. MTF1 and its regulatory gene MT1 are activated by zinc and copper in breast cancer cells in the presence of p53, but not in p53-inactivated cells [65]. In ovarian cancer, the knockdown of MTF1 expression suppressed EMT in ovarian cancer cells [66]. MTF1 is a biomarker for predicting disease recurrence in advanced head and neck cancer [67]. Furthermore, GLS1 is a critical enzyme in glutamine metabolism. There is increasing evidence that the expression level of GLS1 is often raised in tumors and rapidly dividing cells [68–70]. GLS1 catalyzes the conversion of glutamine to glutamate and ammonia nitrogen and is involved in various biological processes [71, 72]. GLS1 is also involved in regulating tumor cell proliferation and migration through multiple pathways [73]. GLS1 is highly expressed in colon cancer, and GLS1 is required for colon cancer cell proliferation and migration [74]. In addition, CDKN2A is a critical tumor suppressor gene and belongs to the cell cycle-dependent kinase inhibitor gene family [75]. It encodes two proteins, including p16INK4A and p14ARF. Both of these proteins can inhibit the occurrence and development of tumors by regulating the cell cycle [75]. Al-Khalaf et al. found that CDKN2A can upregulate the expression of genes related to cell proliferation, such as FGFR1, CCND1, and E2F1 [76]. Previous findings showed that CDKN2A methylation frequency was significantly increased in pancreatic cancer patients and was associated with worse survival in pancreatic cancer patients. CDKN2A methylation plays an essential role in the occurrence and development of pancreatic cancer, and it may become a prognostic marker of pancreatic cancer [77, 78]. The three genes LIPT1, LIAS, and PDHB have not been deeply explored in the field of cancer, especially the mechanism of action in cuprotosis. In future scientific research, we will continue to explore the biological functions of these target genes in the occurrence and development of cancer.

## 5. Conclusions

In this study, we systematically used bioinformatics analysis technology to analyze pancreatic cancer data from TCGA and GTEx. We established a cuprotosis correlation prediction model that was significantly correlated with the prognosis of pancreatic cancer patients. The prediction model included LIPT1, LIAS, and DLAT. Nonetheless, this study still has certain limitations. To improve the reliability of the research results, further clinical experiments are needed to be explored, so we plan to further verify the correlation of this cuprotosis-related prediction model with pancreatic cancer progression through in vivo or in vitro experiments in the future, and in-depth research explores its underlying mechanism in pancreatic cancer.

## Data Availability

The data used to support the findings of this study are available from the corresponding author upon request.

## Abbreviations

TCGA: The Cancer Genome Atlas
GSCA: Gene set cancer analysis
GDSC: Genomics of Drug Sensitivity in Cancer
CTRP: Cancer Therapeutics Response Portal
LASSO: Least absolute shrinkage and selection operator
GSEA: Gene set enrichment analysis
PAAD: Pancreatic adenocarcinoma
GTEx: Genotype-Tissue Expression
TIMER: Tumor Immune Estimation Resource
ROC: Receiver operating characteristic
AUC: Area under curve
FDX1: Ferredoxin 1
LIPT1: Lipoyltransferase 1
LIAS: Lipoic acid synthetase
DLD: Dihydrolipoamide dehydrogenase
DLAT: Dihydrolipoamide S-acetyltransferase
PDHA1: Pyruvate dehydrogenase E1 subunit alpha 1
PDHB: Pyruvate dehydrogenase E1 subunit beta
MTF1: Metal regulatory transcription factor 1
GLS: Glutaminase
CDKN2A: Cyclin-dependent kinase inhibitor 2A
OS: Overall survival
PFS: Progression-free survival
CNV: Copy number variants.
SNV: Single nucleotide variant.
